# Circular RNA circPSEN1 promotes neovascularization by regulating the miR-150-5p/TRIM65 axis in diabetic retinopathy

**DOI:** 10.1016/j.bbrep.2026.102731

**Published:** 2026-08-01

**Authors:** Dongyu Cai, Shouquan Lu, Yujue Wang

**Affiliations:** a Guangzhou Xin Hai Hospital, Guangzhou, 510300, China; b Southern Medical University Laboratory Animal Management Center, Guangzhou, 510515, China

**Keywords:** Diabetic retinopathy, *circPSEN1*, *miR-150-5p*, TRIM65, Neovascularization

## Abstract

Diabetic retinopathy (DR), a major microvascular complication of diabetes, is a leading cause of vision loss. Neovascularization is a hallmark of DR, and circRNA-PSEN1 (circPSEN1) has emerged as a key regulator in its development. However, the precise molecular mechanisms by which circPSEN1 contributes to DR remain unclear. This study investigates the role of circPSEN1 in DR, focusing on its interaction with miR-150-5p and TRIM65. Human retinal microvascular endothelial cells (hRMECs) were cultured under high-glucose (HG) conditions to establish an *in vitro* DR model, and streptozotocin-induced DR rats were used for *in vivo* analysis. Lentivirus-mediated gene modulation was employed to alter expression levels. Cell viability, migration, angiogenesis, and inflammatory cytokine release were assessed using CCK-8, transwell, tube formation assays, and ELISA. Dual-luciferase reporter assays, RNA pull-down, and RNA immunoprecipitation (RIP) techniques were used to examine the interactions between circPSEN1, miR-150-5p, and TRIM65. Elevated circPSEN1 and TRIM65 levels, along with reduced miR-150-5p expression, were observed in retinal tissues from DR rats. In HG-stimulated hRMECs, circPSEN1 knockdown inhibited cell proliferation, migration, and angiogenesis, while reducing inflammatory cytokine release. These effects were reversed by miR-150-5p suppression or TRIM65 overexpression, suggesting a regulatory role of both molecules in DR. Mechanistically, circPSEN1 acts as a sponge for miR-150-5p, enhancing TRIM65 expression and contributing to DR pathogenesis. Our findings indicate that circPSEN1 may serve as a therapeutic target for DR.

## Highlights

• CircPSEN1 is upregulated in diabetic retinopathy and promotes neovascularization.
• Silencing circPSEN1 suppresses endothelial proliferation, migration, and angiogenesis.
• CircPSEN1 functions as a sponge for miR-150-5p, regulating TRIM65 expression.
• The circPSEN1/miR-150-5p/TRIM65 axis drives angiogenesis and inflammation in DR.
• Targeting circPSEN1 may offer a novel therapeutic strategy for diabetic retinopathy.

## 1 Introduction

According to the International Diabetes Federation, diabetes affected 10.5% of adults globally in 2022, with cases expected to climb to 12.2%-impacting approximately 783 million people-by 2045 [1]. Diabetic retinopathy (DR) is a leading microvascular complication of diabetes and the primary cause of vision impairment among working-age adults [2]. As life expectancy increases and socioeconomic development progresses, the global number of patients with DR is expected to increase significantly [3]. Although controlling blood glucose levels and blood pressure, along with laser treatments, can slow the progression of DR, they offer limited benefits in reversing vision loss. The pathogenesis of DR involves complex mechanisms, including oxidative stress, inflammatory cell aggregation, and neovascularization. In recent years, studies on neovascularization factors has significantly advanced, with VEGF-A being extensively studied for its roles in endothelial cell proliferation, retinal neovascularization, and vascular leakage [4]. However, VEGF-A is not the sole factor involved and anti-VEGF-A therapy alone cannot completely resolve neovascularization in proliferative DR [5]. Therefore, further investigations into the mechanisms underlying retinal neovascularization and the identification of key genetic and molecular targets are essential for advancing clinical prevention and treatment strategies for retinal neovascular diseases.

Circular RNAs (circRNAs) are non-coding RNAs abundantly present in eukaryotic cells, generated by back-splicing of precursor mRNAs. Distinct from linear transcripts, circRNAs possess a covalently closed circular structure [6], and they play crucial roles at epigenetic, transcriptional, and translational levels, affecting various aspects of cell biology and human disease development [7,8]. CircRNAs have been associated with diabetes and its complications, suggesting their potential as novel therapeutic targets for these conditions [9]. Several studies have highlighted the role of circRNAs in DR. For instance, circHIPK3 modulates the expression of VEGF, an important vascular endothelial gene, by inhibiting miR-30a-3p, thereby contributing to the development of DR [10]. Additionally, circDNMT3B is down-regulated in DR, leading to retinal microvascular dysfunction by targeting miR-20b-5p [11]. Furthermore, circ-cPWWP2A acts as an endogenous RNA that interacts with miR-579, further upregulating the expression levels of angiopoietin-1 (Ang-1), occludin, and SIRT1, thereby promoting retinal vascular dysfunction and the onset of DR [12]. Emerging evidence from recent transcriptomic analyses has identified a marked elevation of circ_0008521 (circPSEN1) expression in DR patients compared to non-diabetic individuals with vitreous tumors, implicating circPSEN1 as a potential diagnostic indicator for DR [13]. Despite these findings, the functional significance of circPSEN1 in hRMECs under diabetic conditions remains insufficiently characterized and necessitates further investigation.

A growing body of research has established that circRNAs can function as competing endogenous RNAs by binding to common microRNA (miRNA) response elements, thereby effectively sequestering miRNAs and inhibiting their ability to regulate downstream target mRNAs [14,15]. MicRNAs are endogenous RNA molecules approximately 22–23 nucleotides long that function as crucial post-transcriptional regulators, modulating gene expression by binding to target mRNAs. In recent years, numerous miRNAs with essential roles in diverse cellular pathways have been identified across eukaryotic systems [16]. miRNAs are widely distributed in plants, animals, and viruses, and can induce target gene mRNA degradation or translation repression by complementary pairing with the 3′ untranslated region of their target mRNAs. The post-transcriptional regulation of gene expression leads to biological effects by modulating downstream target genes [17]. miR-150-5p levels are significantly downregulated in patients with DR [18]. Evidence suggests that a reduction in miR-150-5p expression levels results in the upregulation of its target genes, which contributes to endothelial cell dysfunction and tubulogenesis *in vitro* in DR [19]. Wang et al. found that linc00174 exacerbated diabetic retinal microvascular damage by influencing the miR-150-5p/VEGFA pathway [20]. Our preliminary analysis using the StarbaseV2.0 database indicated that circ-PSEN1 interacted with miR-150-5p, and that miR-150-5p had clear binding sites in TRIM65. TRIM65, a key E3 ligase that regulates TNRC6A, plays a pivotal role in diabetes [21], and its expression level is significantly elevated in high glucose (HG)-induced hRMECs [22]. Nevertheless, the specific contribution of circPSEN1 in DR need to be fully Inquiried.

Based on this evidence, we propose that circPSEN1 competes with miR-150-5p, thereby increasing TRIM65 expression levels and promoting angiogenesis in hRMECs under a high-sugar environment. Our study aimed to explore the role of the circPSEN1/miR-150-5p/TRIM65 molecular axis in DR with the goal of elucidating the molecular mechanisms driving the onset and progression of DR. Our findings offer a theoretical foundation for the identification of new therapeutic targets for DR.

## 2 Materials and methods

### 2.1 Cell culture and treatment

hRMECs were maintained in endothelial growth medium supplemented with 10% FBS, 100 U/mL penicillin, and 100 μg/mL streptomycin under standard conditions (37°C, 5% CO_2_, humidified atmosphere). Cells were passaged every two days using 0.25% trypsin, and only those in the exponential growth phase were used for experiments. To simulate diabetic retinopathy *in vitro*, hRMECs were exposed to 25 mM glucose for 48 h. Control cells were treated with 5.5 mM glucose supplemented with 19.5 mM mannitol for the same duration to account for osmotic effects.

### 2.2 Cell transfection

Lentiviral vectors targeting circPSEN1 (sh-circPSEN1) and the corresponding negative control (sh-NC), along with miR-150-5p mimics and inhibitors, were obtained from Shanghai Shenggong Biotechnology (China). The cells were transduced as follows: sh-circPSEN1 group: This group was transduced with lentiviral vectors carrying short hairpin RNA (shRNA) specifically targeting circPSEN1 to knock down circPSEN1 expression. sh-NC group: This group was transduced with lentiviral vectors carrying a non-targeting control shRNA (sh-NC) to serve as a negative control. sh-circPSEN1+NC inhibitor group: This group was transduced with sh-circPSEN1 (to knock down circPSEN1) and treated with NC (negative control) inhibitors to assess the effects of circPSEN1 knockdown in the presence of an inhibitor. sh-NC + NC mimic group: This group was transduced with sh-NC (non-targeting control) and treated with NC mimic to evaluate the effects of non-specific knockdown with mimics on the cell behavior. hRMECs at 50%-60% confluency were plated into 12-well plates and transduced accordingly. After 48 h, viral supernatants were harvested, centrifuged at 500 × g for 10 min, filtered, and replaced with fresh culture medium.

### 2.3 Cell counting Kit-8 assay

After 48 h post-transfection, cells were seeded into 96-well plates in triplicate for each group. To evaluate proliferation, 10 μL of MTT solution was added per well, followed by a 2-h incubation at 37°C to allow formazan formation. Subsequently, DMSO was used to dissolve the crystals, and absorbance was recorded at 450 nm using a Bio-Rad microplate reader (Hercules, CA, USA).

### 2.4 Transwell assay

Each group of cells (200 μL suspension) was seeded into the upper chamber of transwell inserts. After 24 h of incubation, cells were fixed in 4% paraformaldehyde for 30 min and stained with 0.1% crystal violet for 10 min. Cells that had migrated to the underside of the membrane were imaged and quantified using a light microscope. Ten randomly selected fields per membrane were imaged, and the number of migrated cells was quantified for statistical analysis.

### 2.5 Tube-formation assay

hRMECs at 70%–80% confluence were detached using trypsin. A 96-well plate was pre-coated with 50 μL Matrigel and incubated for 2 h at 37°C under humidified conditions containing 5% CO_2_ to allow gel polymerization. Subsequently, cells were resuspended to a density of 5 × 10^7^ cells/L, and 100 μL of this suspension was plated onto each Matrigel-coated well. Following an 8-h incubation, tubular network formation was visualized using an inverted microscope. Experiments were conducted in triplicate, and both the number of tubes and total tube length were analyzed using ImageJ software.

### 2.6 Enzyme-linked immunosorbent assay (ELISA)

ELISAs were conducted to determine IL-1β, IL-6, and TNF-α concentrations in hRMECs and retinal tissue. Each 100 μL sample was loaded into the assay wells, washed, and incubated with horseradish peroxidase (HRP)-labeled antibodies for 30 min. Substrate solution was then added, followed by a 15-min incubation at 37°C in the dark. The reaction was terminated with stop solution, and absorbance was recorded at 450 nm.

### 2.7 Dual-luciferase reporter gene assay

Potential binding relationships between miR-150-5p and circPSEN1 or TRIM65 were predicted using StarbaseV2.0. To confirm these predictions, luciferase reporter constructs containing wild-type or mutated sequences of circPSEN1 and TRIM65 (WT-circPSEN1, MUT-circPSEN1, WT-TRIM65, MUT-TRIM65) were engineered. These plasmids were co-transfected into hRMECs alongside either a miR-150-5p mimic or negative control mimic using Lipofectamine™ 2000. Dual-luciferase reporter assays were then performed as per the manufacturer's protocol, with firefly luciferase activity normalized to Renilla luciferase to determine relative signal intensity.

### 2.8 RNA immunoprecipitation (RIP)

RIP was conducted using a commercial kit (Millipore, USA) to validate the interaction between circPSEN1 and miR-150-5p. Cell lysates were prepared in RIP lysis buffer and centrifuged to collect the supernatant. Magnetic beads coupled with either anti-Ago2 or control IgG antibodies were incubated with the lysates overnight at 4°C. After magnetic separation, the immunoprecipitated complexes were treated with proteinase K, and the associated RNA was extracted and quantified via qRT-PCR.

### 2.9 RNA pull-down assay

miR-150-5p probes (Ruibo, China) were mixed with cell lysates and incubated at 25°C for 120 min. Subsequently, the immune complexes were isolated using streptavidin magnetic beads. The beads were then treated with proteinase K buffer for 60 min at 25°C, and the resulting eluted RNA was analyzed using qRT-PCR.

### 2.10 Establishment of a rat model of DR

Male Sprague Dawley (SD) rats (8 weeks old, 240 ± 20 g), sourced from Guangzhou Huaten Biomedical Technology Co., Ltd., were housed under standard laboratory conditions (temperature: 21–25°C; humidity: 60%–70%; 12-h light/dark cycle) with unrestricted access to food and water. Prior to modeling, rats were fasted for 6 h. Diabetic retinopathy (DR) was induced in the experimental group by intraperitoneal injection of streptozotocin (STZ, 50 mg/kg, pH 4.5) for five consecutive days. Control animals received equal volumes of sodium citrate buffer. One week post-injection, blood glucose was measured via tail vein sampling. Rats with glucose levels ≥16.7 mmol/L were classified as diabetic [23]. All the procedures were approved by the Animal Ethics Committee of Guangzhou Huateng Biomedical Technology Co., Ltd. (Approval No.: B202411-11).

### 2.11 Lentivirus transfection

After the successful induction of diabetes (Table 1), 30 SD rats were randomly assigned to one of the following five groups: control, DR, sh-*circPSEN1* + oe-NC, sh-*circPSEN1* + oe-TRIM65, and sh-NC + oe-NC. On day 7 after diabetes induction, fasting blood glucose was measured in all animals by tail-vein sampling before any intravitreal injection was performed. Rats with blood glucose levels ≥16.7 mmol/L were considered diabetic. Subsequently, a total of 30 rats were allocated into five groups, with six rats in each group (n = 6): sham, DR, sh-NC + oe-NC, sh-circPSEN1 + oe-NC, and sh-circPSEN1 + oe-TRIM65. Following completion of the D7 blood glucose measurement and group allocation, the designated groups received an intravitreal injection of 1 μL lentivirus (10^9^ plaque-forming units/mL) later on the same day [24,25]. All subsequent experimental procedures were performed three weeks following lentiviral administration.

**Table 1:** The blood sugar levels of each group of rats.

Group	Pre-modeling (mmol/L)	D7 (mmol/L)	D14 (mmol/L)	D21 (mmol/L)	D28 (mmol/L)
sham	5.4 ± 0.5	5.3 ± 0.4	5.5 ± 0.4	5.3 ± 0.3	5.6 ± 0.6
DR	5.3 ± 0.2	20.2 ± 1.8**	29.8 ± 3.7**	33.3 ± 0.0**	33.2 ± 0.2**
sh-NC + oe-NC	5.5 ± 0.2	21.9 ± 1.1**	31.2 ± 2.1**	33.3 ± 0.0**	33.3 ± 0.0**
sh-circPSEN1+oe-NC	5.4 ± 0.2	20.1 ± 9.1**	30.4 ± 3.8**	33.3 ± 0.0**	33.3 ± 0.0**
sh-circPSEN1+oe-TRIM65	5.4 ± 0.2	28.6 ± 3.3**	27.0 ± 4.0**	33.3 ± 0.0**	33.3 ± 0.0**

**Note:** Data are presented as the mean ± SD (n = 6 per group). D7 blood glucose was measured on day 7 after diabetes induction and **before intravitreal lentiviral injection**. All diabetic animals exceeded the predefined diagnostic threshold of 16.7 mmol/L at D7. One-way ANOVA of the D7 values among the four diabetic groups showed an overall baseline difference (P = 0.023). The relatively higher value in the sh-circPSEN1 + oe-TRIM65 group was verified against the original experimental records and represents a pre-intervention baseline imbalance rather than an effect of lentiviral treatment. **P < 0.01 vs. sham.

### 2.12 Quantitative real-time polymerase chain reaction (qRT-PCR)

RNA was extracted from frozen retinal tissues or cultured cells using TRIzol (Invitrogen). Using 500 ng of RNA, cDNA was synthesized with an ABI kit under the following conditions: 16°C for 30 min, 42°C for 30 min, and 85°C for 5 min. Quantitative PCR was performed utilizing SYBR™ Select Master Mix under the following thermal conditions: initial denaturation at 95°C for 2 min, followed by 40 amplification cycles consisting of 95°C for 15 s and 60°C for 1 min. GAPDH and U6 served as the internal control gene. Relative gene expression levels were analyzed using the 2^−ΔΔCt^ approach. The utilized primer sequences were as follows: circPSEN1, 5′- GAGGACAACCACCTGAGCA-3′ and 5′- TAAGGACCGCAAAGGCTG-3’; miR-150-5p, 5′- TCTCCCAACCCTTGTACCAG-3′ and 5′-GAACATGTCTGCGTATCTC-3’; TRIM65, 5′- AGGAGCAACGCAGTCGGATTGA-3′ and 5′-GCCTGCTTCTTGGCTACCTCTA-3’; GAPDH, 5′-TTGCTTCAGGGTTTCATCCAG-3′ and 5′-GACACTCGCTCAGCTTCTTG-3’; U6, 5′-GCTTCGGCAGCACATATACTAAAAT-3′ and 5′-CGCTTCACGAATTTGCGTGTCAT-3’.

### 2.13 Western blotting

Cells and retinal tissue were lysed using RIPA buffer containing 1% PMSF and placed on ice for 30 min. Protein concentrations were determined via BCA assay. After denaturation at 100°C for 5 min, equal protein amounts were resolved by SDS-PAGE and transferred onto PVDF membranes. Membranes were blocked with 5% TBS for 1 h, then incubated overnight at 4°C with the following primary antibodies: TRIM65 (1:1,000, AP11814c), VEGF (1:1,000, ab32152), ZO-1 (1:1,000, ab276131), occludin (1:500, ab216327), and GAPDH (1:1,000, ab181602). Following triple washing, membranes were treated with HRP-labeled secondary antibody (1:10,000, ab6721) for 1 h at room temperature. Protein bands were visualized by X-ray exposure and quantified using ImageJ software.

### 2.14 Statistical analysis

Statistical processing was carried out utilizing SPSS version 22.0 (IBM Corp., Armonk, NY, USA). Quantitative data were represented as the mean ± standard deviation. To determine differences between two independent datasets, an unpaired two-tailed *t*-test was employed. For analyzing variance among more than two groups, one-way ANOVA was conducted, followed by appropriate post hoc testing where applicable. All experimental procedures were independently repeated three times to ensure reproducibility. A threshold of P < 0.05 was adopted to define statistical significance throughout the analyses.

## 3 Results

### 3.1 Silencing circPSEN1 expression significantly inhibited inflammation and angiogenesis induced by a high glucose concentration in hRMECs

**Fig. 1:**
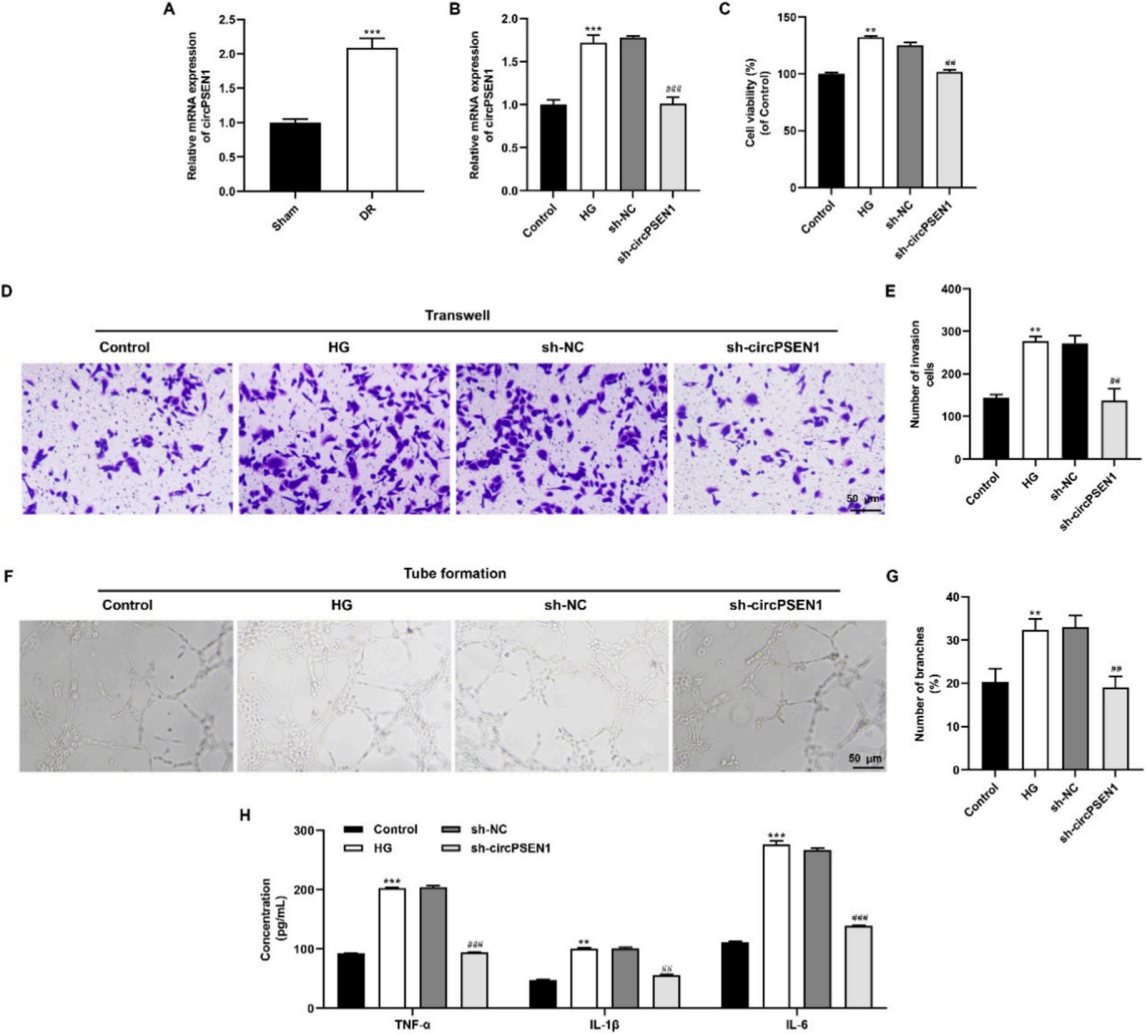
**circPSEN1 knockdown inhibits proliferation, migration, tube formation, and inflammation in high-glucose-treated hRMECs.** (A–B) Relative mRNA expression of circPSEN1 in DR rat retinas and high-glucose (HG)-treated hRMECs. (C) Cell viability measured by CCK-8 assay. (D–E) Representative transwell images and quantification of migrated cells. (F–G) Tube formation and quantification of branch numbers. (H) ELISA quantification of inflammatory cytokines TNF-α, IL-1β, and IL-6 in each group. Data are shown as mean ± SD. ***P < 0.001 vs. control; **P < 0.01 vs. control; ^##^*P* < 0.01, ^###^*P* < 0.001 vs. HG or sh-NC.

To determine whether circPSEN1 is associated with DR, we performed qRT-PCR to detect the expression of circPSEN1 in rats with DR. We found that the expression of circPSEN1 was significantly upregulated (Fig. 1A). Similarly, in HG-treated hRMECs used to simulate the DR cell model, the circPSEN1 expression level was markedly increased (Fig. 1B). Our results suggested that circPSEN1 is implicated in the development of DR. To assess its biological role, we used a vector-based short hairpin RNA (sh-circPSEN1) to silence circPSEN1 expression in HG-treated hRMECs. Cell viability and migratory capacity were assessed using CCK-8 and transwell assays, demonstrating that high-glucose (HG) exposure markedly increased both proliferation and migration of hRMECs (Fig. 1C–E). Tube formation analysis further indicated that HG treatment augmented the angiogenic potential of these cells (Fig. 1F and G). In contrast, knockdown of circPSEN1 significantly attenuated the HG-induced enhancements in cell proliferation, migration, and tube formation. Moreover, ELISA results revealed that HG stimulation elevated the secretion of TNF-α, IL-1β, and IL-6, whereas circPSEN1 silencing substantially diminished these increases (Fig. 1H). Overall, these results indicated that silencing circPSEN1 expression significantly inhibited angiogenesis and inflammation induced in HG-treated HRMECs.

### 3.2 circPSEN1 regulated the expression of mir-150-5p as a sponge in an *in vitro* model of DR

**Fig. 2:**
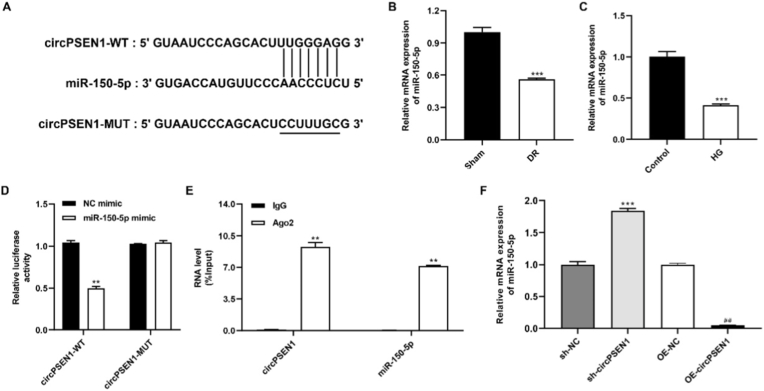
circPSEN1 acts as a molecular sponge for miR-150-5p. (A) Predicted binding sequences between circPSEN1 and miR-150-5p. (B–C) miR-150-5p expression in DR rats and HG-treated hRMECs. (D) Dual-luciferase reporter assay showing direct interaction. (E) RIP enrichment of circPSEN1 and miR-150-5p with Ago2. (F) qRT-PCR analysis of miR-150-5p expression after circPSEN1 knockdown or overexpression. Data are shown as mean ± SD.***P < 0.001 vs. Sham, Control or sh-NC; **P < 0.01 vs. NC mimic or IgG; ^##^*P* < 0.01 vs. OE-NC.

Although circPSEN1 has been implicated in the development of diabetic retinopathy (DR), its regulatory mechanisms remain largely undefined. Using StarBase V2.0, potential binding sites between circPSEN1 and miR-150-5p were predicted (Fig. 2A), suggesting a ceRNA-like interaction. qRT-PCR analysis revealed notably reduced miR-150-5p expression in DR rat retinas and HG-treated hRMECs (Fig. 2B and C). To confirm direct binding, RIP and dual-luciferase assays were performed. RIP showed co-enrichment of circPSEN1 and miR-150-5p with Ago2 (Fig. 2E), and luciferase activity was significantly suppressed in cells expressing wild-type circPSEN1 upon miR-150-5p mimic transfection (Fig. 2D), confirming their interaction. Additionally, circPSEN1 overexpression significantly downregulated miR-150-5p in hRMECs, while silencing circPSEN1 led to upregulated miR-150-5p levels (Fig. 2F). In summary, these results suggested that circPSEN1 negatively regulated miR-150-5p expression in DR.

### 3.3 Downregulation of mir-150-5p reversed the anti-inflammatory and anti-angiogenic effects of circPSEN1 silencing in an *in vitro* model of DR

**Fig. 3:**
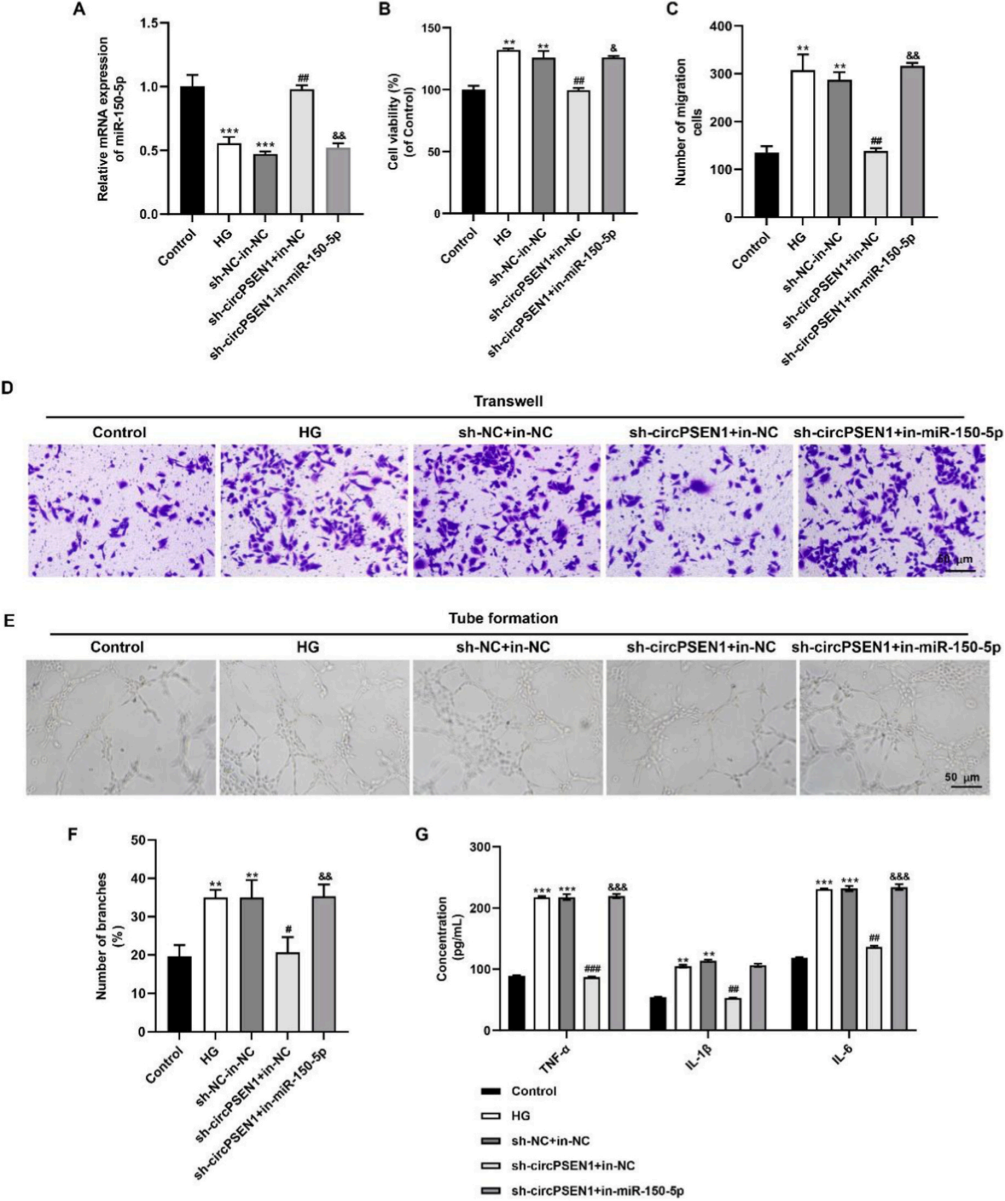
miR-150-5p inhibition reverses the effects of circPSEN1 knockdown on cell function and inflammation. (A) miR-150-5p levels after co-transfection of sh-circPSEN1 and miR-150-5p inhibitors. (B–C) Cell viability and migration. (D–F) Transwell and tube formation assays. (G) ELISA measurement of TNF-α, IL-1β, and IL-6. Data are shown as mean ± SD. ***P < 0.001, **P < 0.01, *P < 0.05 vs. control; ##P < 0.01, #P < 0.05 vs. HG or sh-NC+in-NC; &&P < 0.01, &P < 0.05 vs. sh-circPSEN1+in-NC.

To investigate the regulatory impact of the circPSEN1/miR-150-5p axis on inflammatory and angiogenic responses in hRMECs, cells were co-transfected with sh-circPSEN1 and miR-150-5p inhibitors. RT-qPCR analysis confirmed effective inhibition of miR-150-5p expression (Fig. 3A). HG exposure significantly enhanced cell viability, migration, and tube-forming capacity compared to the negative control (NC) group. In contrast, these cellular functions were markedly attenuated in the sh-circPSEN1+NC inhibitor group relative to the sh-NC + NC mimic group. Notably, transfection with the miR-150-5p inhibitor in the sh-circPSEN1 background restored cell viability, migratory activity, and tube formation compared to the sh-circPSEN1 + NC mimic group (Fig. 3B–F). Furthermore, ELISA results demonstrated reduced concentrations of TNF-α, IL-1β, and IL-6 in the sh-circPSEN1 + NC inhibitor group, whereas these inflammatory cytokines were elevated upon miR-150-5p inhibition in the sh-circPSEN1 background (Fig. 3G). Collectively, these data indicate that circPSEN1 facilitates inflammation and angiogenesis in HG-stimulated hRMECs by suppressing miR-150-5p expression.

### 3.4 miR-150-5p targeted TRIM65 in an *in vitro* model of DR

**Fig. 4:**
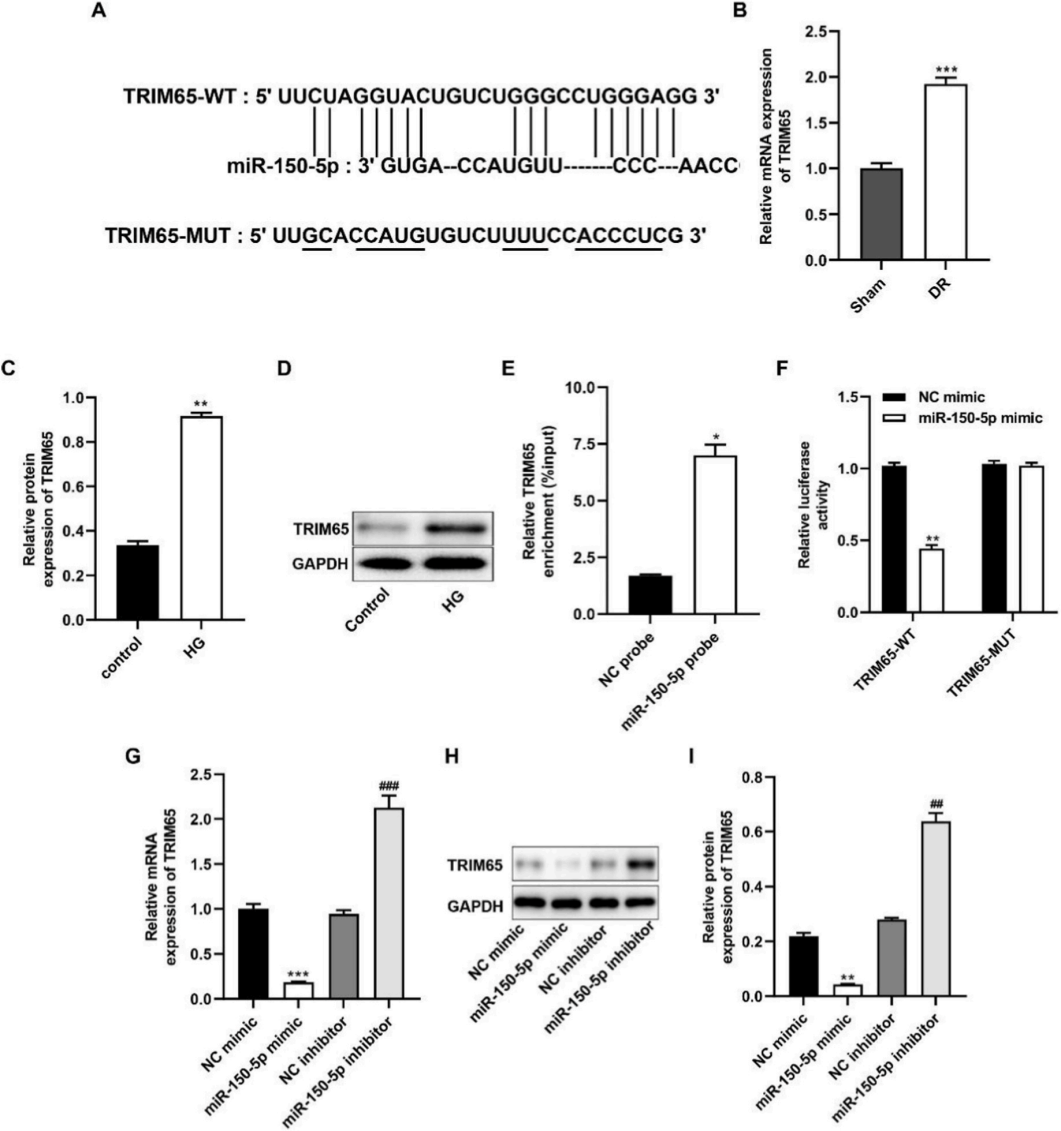
TRIM65 is a direct downstream target of miR-150-5p. (A) Binding sequences between miR-150-5p and TRIM65. (B–C) mRNA and protein levels of TRIM65 in DR retinas and HG-treated hRMECs. (D) Representative Western blot for TRIM65. (E) RNA pull-down assay showing TRIM65 binding to miR-150-5p. (F) Dual-luciferase assay showing direct targeting. (G–I) Effects of miR-150-5p mimic/inhibitor on TRIM65 expression at RNA and protein levels. n = 3, data are shown as mean ± SD. ***P < 0.001, **P < 0.01 vs. control or NC mimic; ^###^*P* < 0.001, ^##^*P* < 0.01 vs. mimic.

We postulated that circPSEN1 modulates TRIM65 expression via miR-150-5p, thereby influencing inflammatory and angiogenic processes in hRMECs. Bioinformatic predictions using StarBase V2.0 identified a putative binding site for miR-150-5p within the TRIM65 transcript (Fig. 4A). Both mRNA and protein analyses demonstrated upregulation of TRIM65 in retinal tissues from DR rats as well as in HG-exposed hRMECs (Fig. 4B–D). To confirm the direct interaction between miR-150-5p and TRIM65, RNA pull-down and dual-luciferase reporter assays were performed. Biotin-labeled miR-150-5p successfully precipitated TRIM65 (Fig. 4E), and co-transfection with the miR-150-5p mimic significantly suppressed luciferase activity in cells expressing wild-type TRIM65 reporter constructs (Fig. 4F). Moreover, overexpression of miR-150-5p in hRMECs resulted in decreased TRIM65 levels, while inhibition of miR-150-5p led to an increase in TRIM65 expression (Fig. 4G–I). Collectively, these results indicate that miR-150-5p directly targets and downregulates TRIM65 in the context of DR *in vitro*.

### 3.5 TRIM65 overexpression reversed the anti-angiogenic and anti-inflammatory effects of CircPSEN1 silencing in an *in vitro* model of DR

**Fig. 5:**
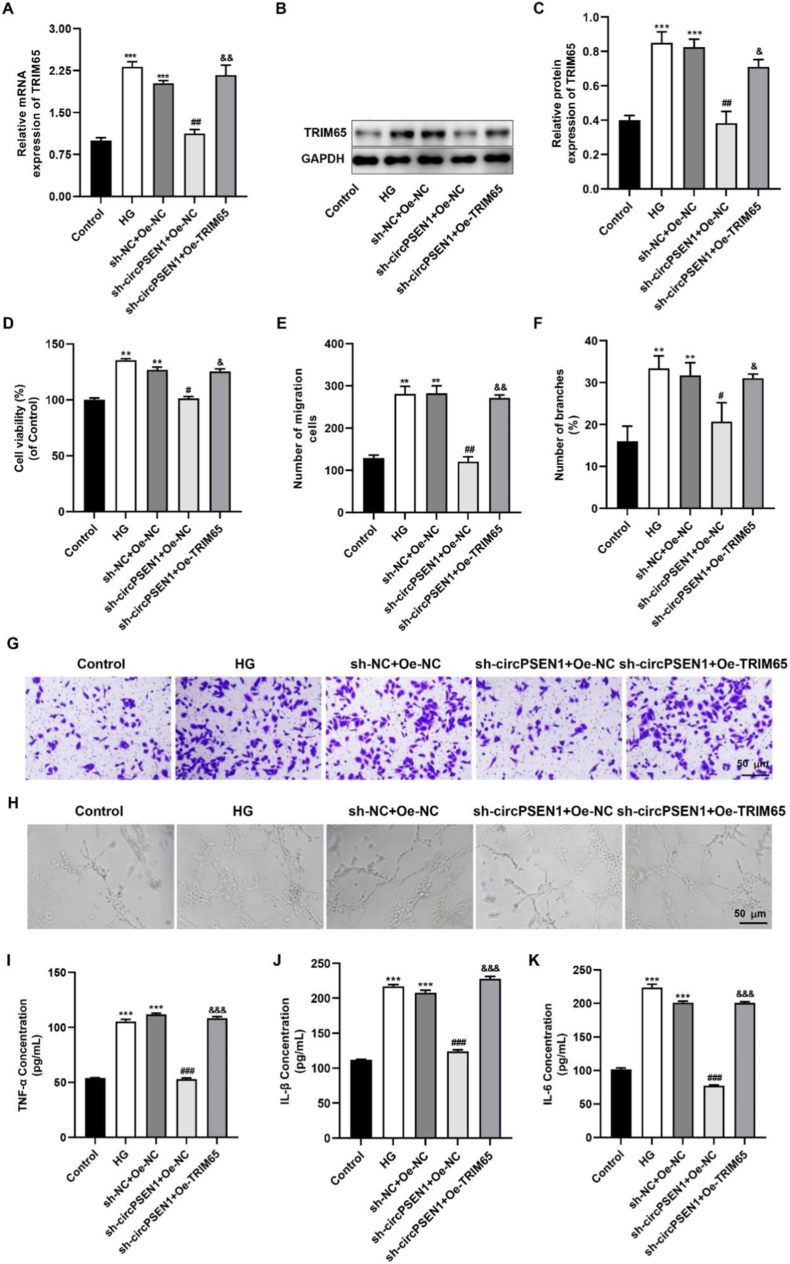
TRIM65 overexpression rescues the inhibitory effects of circPSEN1 knockdown in HG-treated hRMECs. (A–C) TRIM65 mRNA and protein levels after co-transfection. (D) CCK-8 viability assay. (E–F) Transwell migration and tube formation analysis. (G) Representative transwell images. (H) Tube formation assay. (I–K) TNF-α, IL-1β, and IL-6 levels measured by ELISA. Data are shown as mean ± SD. ***P < 0.001, **P < 0.01 vs. control; ^#^*P* < 0.05, ^##^*P* < 0.01 vs. HG or sh-NC + oe-NC; ^&&&^*P* < 0.001, ^&&^*P* < 0.01, ^&^*P* < 0.05 vs. sh-circPSEN1+oe-NC.

To investigate whether circPSEN1 affects inflammation and angiogenesis in hRMECs by regulating the miR-150-5p–TRIM65 molecular axis, we transfected HG-treated hRMECs with sh-circPSEN1 or oe-TRIM65. Transfection with sh-circPSEN1 decreased TRIM65 expression levels. When sh-circPSEN1 and oe-TRIM65 were co-transfected into hRMECs, TRIM65 mRNA and protein expression levels were partially restored (Fig. 5A–C). Furthermore, overexpression of TRIM65 counteracted the inhibitory effects of circPSEN1 knockdown on cell proliferation, migration, angiogenesis, and inflammatory cytokine production in HG-stimulated hRMECs (Fig. 5D–K). These results suggest that circPSEN1 promotes angiogenic and inflammatory responses in the *in vitro* DR model by elevating TRIM65 expression.

### 3.6 CircPSEN1 promoted retinal inflammation and vascular dysfunction in rats with DR through the mir-150-5p/TRIM65 axis

**Fig. 6:**
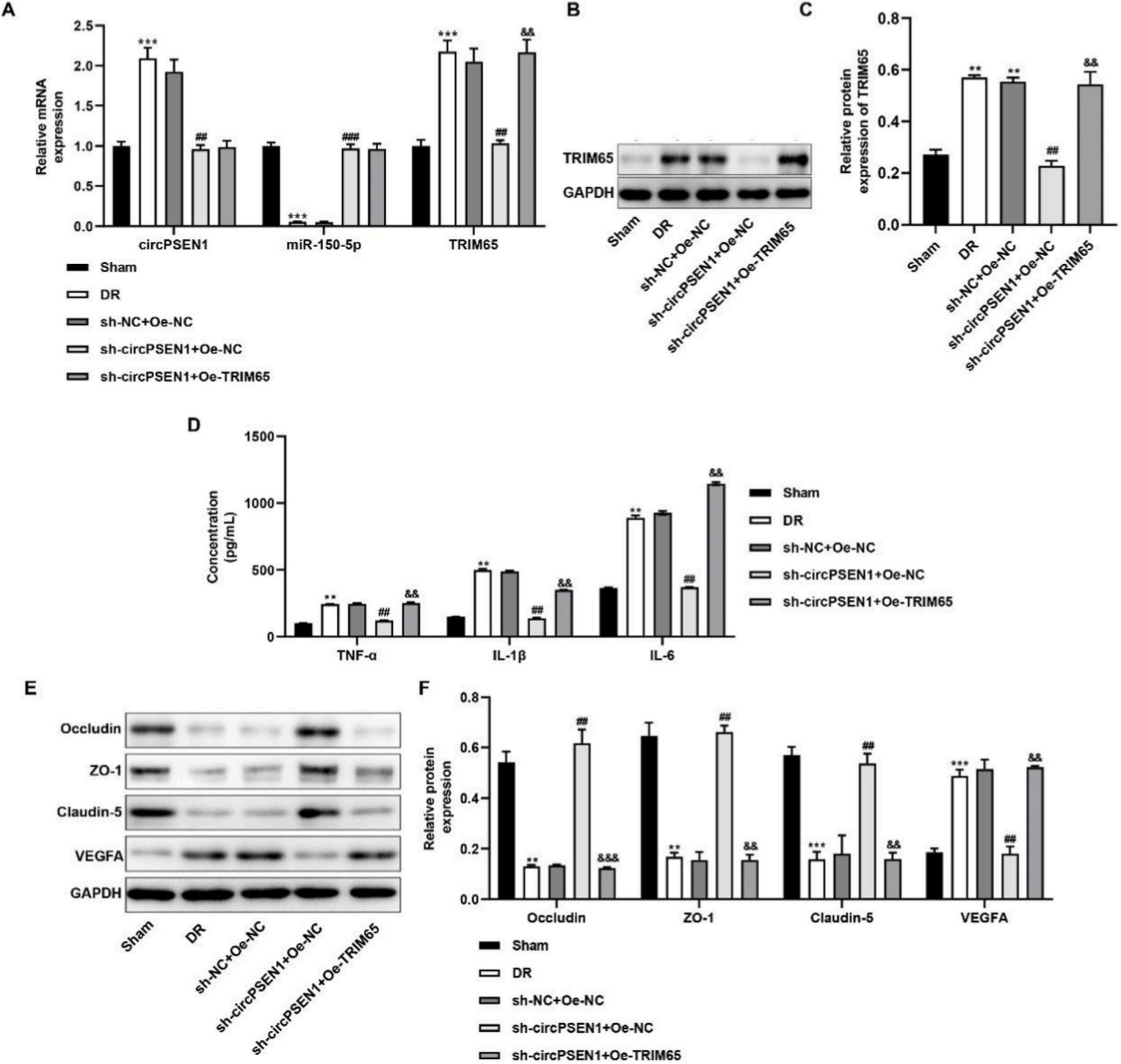
circPSEN1 modulates the miR-150-5p/TRIM65 axis to promote retinal inflammation and vascular dysfunction *in vivo*. (A–C) Retinal expression levels of circPSEN1, miR-150-5p, and TRIM65 in each group. (D) Quantification of TNF-α, IL-1β, and IL-6 by ELISA. (E–F) Western blotting and quantitative analysis of VEGF-A and blood–retinal barrier-related tight junction proteins, including Occludin, ZO-1, and Claudin-5, in retinal tissues. Data are presented as the mean ± SD. ***P < 0.001 and **P < 0.01 vs. Sham; ##P < 0.01 vs. DR or sh-NC + oe-NC; &&&P < 0.001 and &P < 0.05 vs. sh-circPSEN1 + oe-NC.

To further investigate the role of the circPSEN1/miR-150-5p/TRIM65 axis in DR *in vivo*, we established a rat model of DR and injected lentiviruses carrying sh-circPSEN1 + oe-NC, sh-circPSEN1 + oe-TRIM65, or sh-NC + oe-NC into the vitreous bodies of the rats. Our findings revealed an increase in circPSEN1 and TRIM65 expression levels and a decrease in miR-150-5p expression levels in the retinal tissues of rats with DR (Fig. 6A–C). ELISAs showed that retinal tissues from rats with DR exhibited significantly elevated levels of pro-inflammatory cytokines, including IL-6, TNF-α, and IL-1β. Injection of sh-circPSEN1 led to reduced levels of circPSEN1 and TRIM65, increased miR-150-5p expression, and decreased levels of pro-inflammatory cytokines(Fig. 6D). Notably, overexpression of TRIM65 (oe-TRIM65) reversed the inhibitory effects of sh-circPSEN1 on TRIM65 expression, and retinal inflammation. Western blotting analysis showed that VEGF-A expression levels were significantly increased in the retinas of rats with DR, whereas key proteins involved in blood-retina barrier integrity, such as Occludin, ZO-1, and Claudin-5, which are crucial for the maintenance of endothelial tight junctions, were downregulated. These proteins are essential for regulating endothelial permeability, and their disruption contributes to the increased vascular leakage observed in DR [[26], [27], [28]]. Silencing of circPSEN1 resulted in a reduction in VEGF-A levels and an increase in the expression levels of these permeability-related proteins, including Occludin, ZO-1, and Claudin-5, suggesting an improvement in blood-retina barrier function. These changes were reversed by TRIM65 overexpression (Fig. 6E–F). In conclusion, the circPSEN1/miR-150-5p/TRIM65 molecular axis contributed to retinal inflammation and vascular dysfunction in rats with DR, as evidenced by alterations in inflammatory cytokines, VEGF-A expression, and blood-retinal barrier-related proteins.

## 4 Discussion

DR, a prevalent microvascular complication of diabetes, is marked by retinal vascular impairment as a central pathological hallmark. Current estimates suggest that nearly one-third of individuals with diabetes will eventually develop DR. As both the incidence of diabetes and patient longevity continue to rise, the global population affected by DR is anticipated to reach 629 million by 2045 [29,30]. While DR is not acutely life-threatening, sustained hyperglycemia leads to progressive damage of the retinal microvasculature, resulting in serious complications such as diabetic macular edema, vitreous hemorrhage, and retinal detachment. These sequelae often culminate in irreversible vision loss and profoundly reduce the quality of life for affected individuals [31]. Current clinical strategies for managing DR include controlling blood glucose, blood pressure, along with localized treatments, such as anti-inflammatory therapies, anti-VEGF drugs, laser photocoagulation, and vitrectomy [32]. VEGF is considered the primary factor driving neovascularization in DR, and the advent of intravitreal anti-VEGF therapies has significantly improved treatment outcomes [33]. In this study, we revealed the upregulation of *circPSEN1* and TRIM65, along with the downregulation of miR-150-5p in rats with DR. Our results demonstrate that circPSEN1 facilitates TRIM65 upregulation through competitive interaction with miR-150-5p, thereby enhancing neovascularization in diabetic retinopathy. These data underscore the potential of targeting the circPSEN1/miR-150-5p/TRIM65 axis to inhibit pathological angiogenesis associated with DR.

There have been significant advances in understanding the factors that contribute to neovascularization, with VEGF-A being a central focus because of its role in promoting retinal neovascularization and vascular leakage [34]. For example, in DR rat models, disruption of blood flow autoregulation via TRPV2 activation recapitulates key pathological features of the disease [35]. Additionally, eIF4A3 modulates the expression of circEHMT1, thereby influencing endothelial cell injury and neovascularization in DR [36]. Several miRNAs-including miR-205-5p, miR-23a, and miR-15b have also been identified as critical regulators of VEGF expression in retinal endothelial cells, highlighting their pivotal roles in the angiogenic processes associated with DR [[37], [38], [39]]. We identified miR-150-5p as an indirect regulator of VEGFA in hRMECs. Although the role of miR-150-5p in hRMECs remains underexplored, accumulating evidence suggests its regulatory function in endothelial biology. Notably, miR-150-5p has been shown to alleviate oxygen-glucose deprivation-induced injury in brain microvascular endothelial cells by downregulating MLLT1 expression [40]. Additionally, miR-150-5p has been shown to play a role in acute pulmonary embolism and pulmonary hypertension, as well as in regulating ox-LDL-induced endothelial cell injury [41]. Moreover, decreased miR-150-5p expression levels activate the SIRT1/p53/AMPK signaling pathway, improving diabetic nephropathy, thus highlighting its role in the regulation of endothelial function. Furthermore, lncRNA-miRNA interactions are critical for the development of DR [42]. Based on bioinformatics predictions and experimental validation, we established the circPSEN1/miR-150-5p molecular axis in hRMECs, contributing to our understanding of retinal vascular dysfunction in DR.

Recent research indicates that circPSEN1 is involved in the regulation of ferroptosis, with its suppression via the miR-200b-3p/cofilin-2 pathway offering protective effects in high-glucose-stimulated retinal pigment epithelial cells [13]. Furthermore, circPSEN1 has been shown to promote autophagy-dependent ferroptosis in respiratory epithelial cells subjected to environmental toxins such as carbon black and cadmium [43]. Despite these advances, the specific functions and mechanistic contributions of circPSEN1 in DR have not been fully elucidated. In the present study, we observed that knockdown of circPSEN1 inhibits cell ability, angiogenesis, and pro-inflammatory cytokine release in HG-exposed hRMECs. Increasing evidence supports the regulatory roles of circRNA/miRNA networks in mRNA expression within the context of DR; for instance, circCOL1A2 modulates angiogenesis via the miR-29b/VEGF pathway [44], and circZNF532 regulates both angiogenesis and inflammation through the miR-1243/CARM1 axis [45]. However, the function of the circPSEN1/miR-150-5p/TRIM65 pathway in hRMECs has not previously been investigated. Our findings reveal that silencing circPSEN1 markedly attenuates inflammatory and angiogenic responses in HG-treated hRMECs, effects that are counteracted by either miR-150-5p inhibition or TRIM65 overexpression. These findings were further supported by *in vivo* experiments showing that circPSEN1 contributes to retinal inflammation and vascular barrier impairment in DR rats. Compromise of the blood–retinal barrier (BRB) is a hallmark of DR, leading to increased vascular permeability and progressive vision impairment [46,47]. The BRB relies on the integrity of tight and adherens junctions, maintained by transmembrane proteins including claudins, occludins, and ZO-1, which regulate endothelial permeability [27]. In our *in vitro* model, circPSEN1 silencing reduced the levels of inflammatory cytokines and VEGFA, while enhancing the expression of tight junction proteins ZO-1, claudin-5, and occludin-effects that were reversed by TRIM65 overexpression. Collectively, our results demonstrate that circPSEN1 facilitates inflammatory responses in DR through modulation of the miR-150-5p/TRIM65 axis. This regulatory pathway offers novel insight into the molecular pathogenesis of DR and identifies circPSEN1 as a potential therapeutic target for mitigating disease progression and preserving vision.

Several limitations should be acknowledged in this study. First, our study primarily focused on human retinal microvascular endothelial cells (hRMECs), which play a central role in retinal neovascularization and vascular dysfunction during diabetic retinopathy [[48], [49], [50]]. Although we demonstrated that the circPSEN1/miR-150-5p/TRIM65 axis regulates pathological responses in hRMECs under diabetic conditions, DR is a complex neurovascular disorder involving multiple retinal cell types. Other retinal cells, including Müller cells and retinal pigment epithelial (RPE) cells, are also essential for maintaining retinal homeostasis and are actively involved in inflammation [51], oxidative stress, and retinal degeneration during DR progression [52]. Therefore, whether the circPSEN1/miR-150-5p/TRIM65 pathway exerts similar regulatory effects in these retinal cell populations remains to be further investigated. Second, although exposure to 25 mM glucose for 48 h has been widely used in cellular models of hyperglycemia-induced oxidative stress, mitochondrial dysfunction, and metabolic injury, we did not perform comprehensive dose-response or time-course experiments to determine whether different glucose concentrations or exposure durations could induce distinct biological effects. The glucose concentration and 48-h treatment duration selected in the present study were based on previous reports demonstrating that this high-glucose exposure protocol is sufficient to induce cellular stress responses *in vitro* [[53], [54], [55]]. Nevertheless, future studies incorporating multiple glucose concentrations and longitudinal analyses will be necessary to further characterize the dynamic regulation of the circPSEN1/miR-150-5p/TRIM65 axis and determine its physiological relevance under varying diabetic conditions. In addition, a transient baseline glycemic imbalance was observed among the diabetic groups at D7 before intravitreal lentiviral injection. Although all diabetic animals met the predefined hyperglycemia criterion and exhibited sustained hyperglycemia during the subsequent observation period, with broadly comparable blood glucose levels at the final D28 endpoint when retinal tissues were collected, the possibility that the initial glycemic imbalance may have influenced the subsequent retinal and molecular outcomes cannot be completely excluded. Future studies should consider stratified randomization based on baseline blood glucose levels to minimize intergroup glycemic imbalance.

In conclusion, our study is the first to reveal the involvement of circPSEN1 and miR-150-5p in angiogenesis in DR. Our research highlights the regulatory role of the circPSEN1/miR-150-5p axis in controlling TRIM65 expression, offering new insights that may contribute to the clinical treatment of DR. We found that circPSEN1 promotes TRIM65 expression by suppressing miR-150-5p, thereby exacerbating inflammatory responses and vascular dysfunction during DR progression. These results highlight circPSEN1, miR-150-5p, and TRIM65 as promising molecular targets for the development of novel anti-DR therapies. Nevertheless, this study has certain limitations. The relatively small sample size may restrict the generalizability of the findings. Additionally, given that TRIM65 functions as an E3 ubiquitin ligase known to regulate TNRC6A, further investigation into its downstream signaling cascades in the context of DR is warranted. Future studies should also include comprehensive preclinical evaluations to facilitate the translation of these molecular insights into effective anti-angiogenic treatments for diabetic retinopathy.

## CRediT authorship contribution statement

**Dongyu Cai:** Conceptualization, Data curation, Formal analysis, Writing – original draft. **Shouquan Lu:** Investigation, Methodology, Project administration. **Yujue Wang:** Data curation, Writing – original draft, Writing – review & editing.

## Ethics declaration

This study was conducted in accordance with the ARRIVE (Animal Research: Reporting of *In vivo* Experiments) guidelines. This study was approved by the Animal Ethics Committee of Guangzhou Huateng Biomedical Technology Co., Ltd. (Approval No. B202411-11)

## Declaration of generative AI and AI-assisted technologies in the writing process

During the preparation of this work, generative AI and AI-assisted technologies are not used.

## Funding

Not applicable.

## Declaration of competing interest

The authors declare that they have no known competing financial interests or personal relationships that could have appeared to influence the work reported in this paper.

## Data Availability

Data will be made available on request.
